# Pulsed Light Stimulation Increases Boundary Preference and Periodicity of Episodic Motor Activity in *Drosophila melanogaster*

**DOI:** 10.1371/journal.pone.0163976

**Published:** 2016-09-29

**Authors:** Shuang Qiu, Chengfeng Xiao, R. Meldrum Robertson

**Affiliations:** Department of Biology, Queen’s University, Kingston, Ontario, Canada, K7L 3N6; Universitat Regensburg, GERMANY

## Abstract

There is considerable interest in the therapeutic benefits of long-term sensory stimulation for improving cognitive abilities and motor performance of stroke patients. The rationale is that such stimulation would activate mechanisms of neural plasticity to promote enhanced coordination and associated circuit functions. Experimental approaches to characterize such mechanisms are needed. *Drosophila melanogaster* is one of the most attractive model organisms to investigate neural mechanisms responsible for stimulation-induced behaviors with its powerful accessibility to genetic analysis. In this study, the effect of chronic sensory stimulation (pulsed light stimulation) on motor activity in *w*^*1118*^ flies was investigated. Flies were exposed to a chronic pulsed light stimulation protocol prior to testing their performance in a standard locomotion assay. Flies responded to pulsed light stimulation with increased boundary preference and travel distance in a circular arena. In addition, pulsed light stimulation increased the power of extracellular electrical activity, leading to the enhancement of periodic electrical activity which was associated with a centrally-generated motor pattern (struggling behavior). In contrast, such periodic events were largely missing in *w*^*1118*^ flies without pulsed light treatment. These data suggest that the sensory stimulation induced a response in motor activity associated with the modifications of electrical activity in the central nervous system (CNS). Finally, without pulsed light treatment, the wild-type genetic background was associated with the occurrence of the periodic activity in wild-type Canton S (CS) flies, and *w*^*+*^ modulated the consistency of periodicity. We conclude that pulsed light stimulation modifies behavioral and electrophysiological activities in *w*^*1118*^ flies. These data provide a foundation for future research on the genetic mechanisms of neural plasticity underlying such behavioral modification.

## Introduction

Neuroplasticity is the ability of the brain to adapt to, or to be modified by, environmental stress, injury or trainings [1–4]. Sensorimotor dysfunctions caused by cerebral impairment have many physiological and mental complications, which are ideal for the evaluation of brain plasticity. Several motor rehabilitation techniques have been developed, such as constraint-induced movement therapy (CIMT), in which task-specific training is applied to the paretic hand to force neural reorganization and improve motor function [5]. Recently, many attempts have been focused on therapeutic effects of repetitive stimulation. It has been shown that repetitive stimulation has positive effects on motor activity and cognitive abilities in healthy subjects [6–8], and also patients with chronic stroke [9, 10] or cerebral lesions [11]. However, the detailed mechanisms underlying the mitigation of brain damage by repetitive stimulation are still elusive. Understanding these mechanisms would consolidate the procedures and their future application in promoting brain recovery.

*Drosophila melanogaster* exhibits extensive behavioral plasticity. Several fly models of genetic diseases and neurodegenerative disorders have been established, such as for Parkinson's disease [12], Huntington's disease [13], and Alzheimer’s disease [14]. These models are proven to be important tools for identification of novel genes and molecules as therapeutic targets. In addition, flies provide an ideal model to study cell apoptosis [15], immune responses [16], sleep [17], and pharmacological screens [18, 19]. With its powerful accessibility to genetic analysis, *Drosophila* is also one of the most outstanding animal models to study brain functions [20]. Flies can be trained to improve behavioral performance including learning, courtship, memory, and odor avoidance [21–24]. The training approach can use odor, heat, electrical stimulation, or a single touch of an appendage between flies [21, 24–26]. Such a strong plasticity in the fly offers great opportunities to explore the contributions of candidate genes to the development of motor and neural adaptations by external repetitive stimulations.

The effect of chronic sensory stimulation on motor activity in the animal model of *Drosophila* was examined because of the potential to use rapid molecular genetic techniques for investigating mechanisms underlying how stimulation facilitates recovery from brain injury. *w*^*1118*^ flies, which are widely used as an isogenic background for producing transgenic flies, have a null mutation of the *white* gene and have a number of locomotor, neurological and cognitive abnormalities or alterations [27–31]. *w*^*1118*^ flies display locomotor impairment with reduced boundary preference compared with wild type Canton S (CS) flies, however, this impairment is unlikely associated with *white* mutation [32]. In *Drosophila*, sensory inputs can induce coherent potential waves in the brain [33–36] and in this study, a pulsed light stimulation protocol was established based on the fact that *w*^*1118*^ lack eye pigmentation and their photoreceptors receive around 19 times more light than those of wild-type flies [29, 37]. This would enhance the effect of the stimulation and could result in more effective plasticity.

We examined the effects of pulsed light stimulation on *w*^*1118*^ flies at both the level of behavior and the level of electrical activity which was associated with a centrally-generated motor pattern. The motor activity in a locomotor assay was investigated by measuring the boundary preference and distance travelled in a circular arena. The effects of stimulation on the electrical activity associated with the centrally-generated struggling motor pattern were examined by measuring the power of extracellular electrical activity. Also, without pulsed light treatment, the mechanism of the periodic electrical activity associated with the centrally-generated motor pattern which occurred in wild type CS flies was investigated as well.

## Materials and Methods

### Flies

Wildtype CS (Bloomington stock center) and mutant *w*^*1118*^ strains (L. Seroude laboratory, Queen’s University) used for the study were raised with standard medium (0.01% molasses, 8.2% cornmeal, 3.4% killed yeast, 0.94% agar, 0.18% benzoic acid, 0.66% propionic acid) at room temperature 21–23°C, 60–70% humidity. A 12h/12 h light/dark cycle was provided by three light bulbs (Philips 13 W compact fluorescent energy saver) with lights on at 7 am and off at 7 pm. Male flies were collected within 2 days after eclosion and raised for at least 3 days free of nitrogen paralysis before the recording and locomotor assay. All experiments were performed between 10 am and 4 pm during the daytime.

Progeny flies (F1 and F10) were prepared by single cross or serial backcrossing between CS and *w*^*1118*^ as described previously [32]. Briefly, for generating F10, a male CS or *w*^*1118*^ was initially crossed into *w*^*1118*^ or CS virgin females to have two different *white* alleles (*w*^*+*^ and *w*^*1118*^) in first generation (F1) heterozygous flies. These females were then backcrossed with *w*^*1118*^ or CS strain for nine consecutive generations. Two resulting fly lines were established: *w*^*+*^ (*w*^*1118*^), which were red-eyed and carried the *w*^*+*^ allele in isogenic first chromosome together with isogenic second and third chromosomes, with the *w*^*1118*^ cytoplasmic background; and *w*^*1118*^ (CS), which were white-eyed and carried the *w*^1118^ allele in wildtype first chromosome together with wildtype second and third chromosomes, with the wildtype cytoplasmic background.

### Pulsed light stimulation

Groups of *w*^*1118*^ flies were subjected to pulsed light stimulation (continuous cycles of 5 s ON– 15 s OFF) of white light (Rxment® 5050 SMD LED light strip) during the 12 h daytime, followed by 12 h dark for entire life cycle. They were then collected within 2 days after emergence and raised for four additional days free of anoxic exposure in the pulsed/dark condition. Before loading the flies into the arenas and starting the experiments, a period of 1 h was allowed for the pulsed flies to adapt to the experimental conditions.

### Locomotor assay

Based on a previously described protocol, the locomotor assay was conducted at room temperature 21–23°C by using a white light box (Logan portaview slide/transparency viewer) with a 5000 K color-corrected fluorescent lamp [32]. Individual flies were restrained in a circular arena (1.27 cm in diameter and 0.3 cm in depth). The locomotion was video-captured and analyzed with written scripts using Open Computer Vision 2.0 (OpenCV2.0). After a 5 min adaptation in the arena, the locomotor parameters including percent time on perimeter (% TOP) over a period of 60 s, travel distance within first 20 s and 0.2 s path increments were examined between different groups of flies. It has been shown that % TOP per min is maintained at steady levels without decline for five consecutive minutes [32], indicating that the selection of first 60 s for evaluating % TOP or 20 s to calculate travel distance and 0.2 s path increments could all be considered to represent long-term behavior.

### Electrophysiology

Extracellular recording was performed during the daytime (10 am to 4 pm) under regular light illumination. An individual fly was secured in a trimmed pipette tip (200 μl) with head exposed while the thorax and abdomen were restrained inside the tip. A small amount of wax was applied underneath the head to limit its movement. An incision along the dorsal ridge between compound eyes was made to allow a glass electrode filled with 1 M potassium acetate (5–10 megaohms) to be inserted to the middle brain. The extracellular potential was recorded against a reference electrode (Ag/AgCl wire) placed in fly thorax. Electrical signals were acquired using AxoScope 10 software (Molecular Devices) with a pH/ION amplifier (Model 2000, A-M Systems) and a digitizer (Digidata 1550A, Molecular Devices) at 1 KHz. A 5 min period was allowed for the fly preparation to recover from tissue penetration. Preparations with large spontaneous direct -current (DC) potential shifts indicating spreading depolarization [38], were rejected. 20 min extracellular electrical activity was recorded because spontaneous DC potential shifts often occurred after 20 min as the preparation deteriorated (data not shown). The power spectrum and auto-correlation analyses were conducted with Clampfit 10 software (Molecular Devices).

### Statistics

Fisher’s exact tests and Chi-square tests were performed to examine the association between genetic contributions and autocorrelation estimates of rhythmic motor activities. D’Agostino & Pearson omnibus normality test was conducted to examine the data distribution. Because part of the data have non-Gaussian distribution, the nonparametric Mann-Whitney test or Kruskal-Wallis test with post comparison was performed to examine the difference of medians between groups. A *P* <0.05 was considered as indicating statistical significance.

## Results

### Pulsed light stimulation increased the time spent on perimeter in *w*^*1118*^ flies

**Fig 1:**
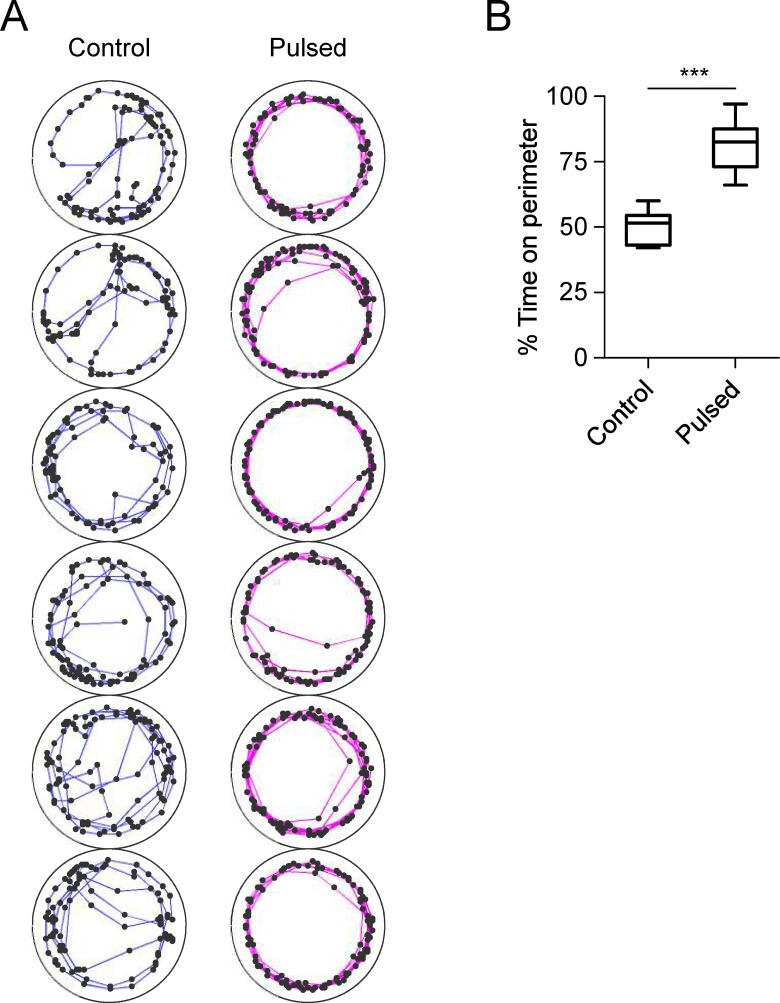
Increased boundary preference in *w*^*1118*^ male flies after pulsed light stimulation. (A) Locomotor trajectories in the circular arenas. Each circle represents the arena (1.27cm diameter). The dots are the fly positions (calculated centers of mass) and the connecting lines show the trajectories during 20 s of locomotion for control (blue lines) and pulsed flies (pink lines). (B) % TOP in control (n = 8) and pulsed (n = 8) *w*^*1118*^ flies. Asterisks (***) indicate *P* < 0.001 by Mann-Whitney test.

To examine the consequence of pulsed light stimulation at behavioral level, we analyzed locomotor activity of *w*^*1118*^ flies in the circular arenas (1.27 cm diameter) by following the reported assay [32]. Throughout a 20 s period, control flies walked and turned actively in the arenas. Each fly showed a preference for staying on the perimeter and also a substantial probability of crossing the central region of the arena (Fig 1A). After pulsed light stimulation, *w*^*1118*^ flies displayed a strong preference for the perimeter. % TOP in *w*^*1118*^ flies with pulsed light stimulation (median 82.5%, interquartile range (IQR) 73.0–87.5%) was higher than that in *w*^*1118*^ controls (median 51.5%, IQR 43.0–54.5%) (*P* < 0.001, Mann-Whitney test) (Fig 1B). Therefore, pulsed light stimulation increased boundary preference during locomotion.

### Pulsed light stimulation increased 0.2 s path increments and travel distance within 20 s in *w*^*1118*^ flies

**Fig 2:**
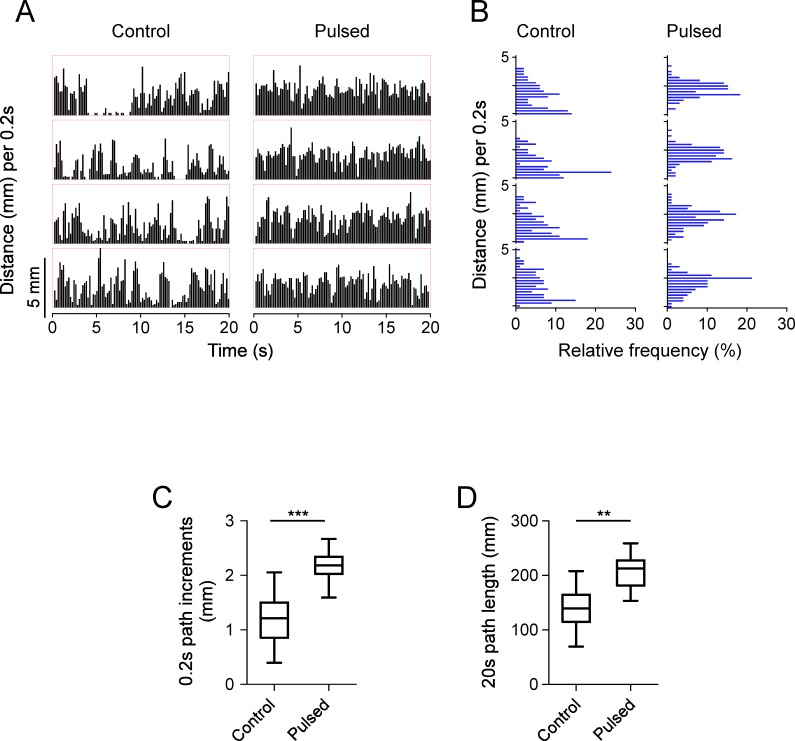
Increased path increments and travel distance in *w*^*1118*^ male flies after pulsed light stimulation. (A) Distances travelled every 0.2 s are plotted during 20 s of locomotion. (B) Relative frequency (%) of the 0.2 s path increments (with bin widths of 0.25 mm) in control and pulsed *w*^*1118*^ flies. (C) 0.2 s path increments in control (n = 8) and pulsed (n = 8) *w*^*1118*^ flies. Asterisks (***) indicate *P* < 0.001 by Mann-Whitney test. (D) 20 s path length in control (n = 8) and pulsed (n = 8) *w*^*1118*^ flies. Asterisks (**) indicate *P* < 0.01 by Mann-Whitney test.

We further examined locomotor performance by comparing 0.2 s path increments in control and pulsed *w*^*1118*^ flies. *w*^*1118*^ control flies walked intermittently with large variance of step size, whereas pulsed flies walked with few stops and relatively consistent step size (Fig 2A). The relative frequency (%) of the 0.2 s path increments (with a bin width of 0.25 mm) was calculated (Fig 2B). Pulsed flies traveled with the 0.2 s path increments (2.2 mm, IQR 2.0–2.3 mm) larger than controls (1.2 mm, IQR 0.9–1.5 mm) (*P* <0.05, Mann-Whitney test) (Fig 2C). The 0.2 s path increments of pulsed flies were consistent with the average walking speed of Oregon-R flies, which is within the range of 1.44–8.94 mm/0.2 s [39]. In addition to the differences of walk-stop performance and 0.2 s path increments, pulsed flies clearly traveled longer distances (median 212.9 mm, IQR 181.5–227.6 mm) than control flies (median 139.5 mm, IQR 114.9–164.6 mm) (*P* <0.05, Mann-Whitney test) (Fig 2D), which is consistent with the previous report showing that flies increase step size and stepping frequency simultaneously to increase walking speed (travel distance within a period of time) [40]. Thus pulsed light stimulation modified walk-stop performance, 0.2 s path increments and travel distance within 20 s.

### Reduced periodicity of electrical activity associated with a centrally-generated motor pattern in *w*^*1118*^ flies without pulsed light stimulation

**Fig 3:**
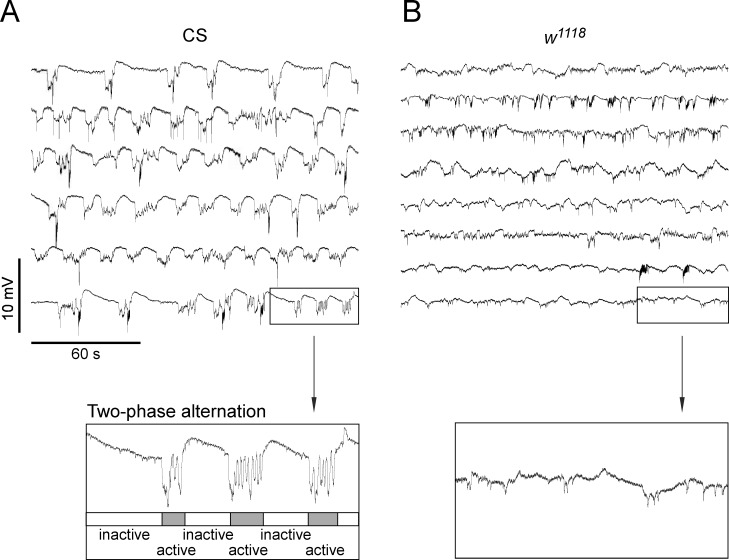
Reduced periodicity of centrally-generated motor pattern in *w*^*1118*^ male flies without pulsed light treatment. (A) Extracellular recordings in the middle brain in wild-type CS male flies without pulsed light treatment. Each trace represents the recording from a single fly. The inset shows a typical alternation of inactive/active phases (with relatively longer duration for inactive phase). (B) Recordings from the mutant *w*^*1118*^ male flies without pulsed light treatment. Inset indicates the apparent loss or reduction of two-phase alternation of extracellular electrical activity.

Restrained adult CS flies express an episodic motor activity which may reflect struggling behavior in an attempt to free themselves (S1 Video), and for which the underlying motor pattern can be recorded extracellularly. It shows that the electrical activity was always associated with the struggling activity from three separate experiments. Without pulsed light treatment, CS fly brains displayed rhythmic deflections with the periodicity of ~19 s (Fig 3A), which were clearly accompanied with the struggling movement (S1 Video). This episodic motor activity was observed consistently in individual CS flies. During 20 min recording, such activity showed a two-phase alternation: inactive phase and active phase. During the inactive phase, the fluctuations were relatively small in amplitude and the baselines were quite stable, whereas during the active phase, the activity showed large fluctuations (Fig 3A inset). On the other hand, the struggling activity in *w*^*1118*^ flies (without pulsed light treatment) had the features of small amplitude and continuous fluctuations with no apparent rhythmicity (Fig 3B), and the two-phase alternation was diminished (Fig 3B inset).

### Pulsed light stimulation increased the electrical activity associated with struggling motor activity in *w*^*1118*^ flies

**Fig 4:**
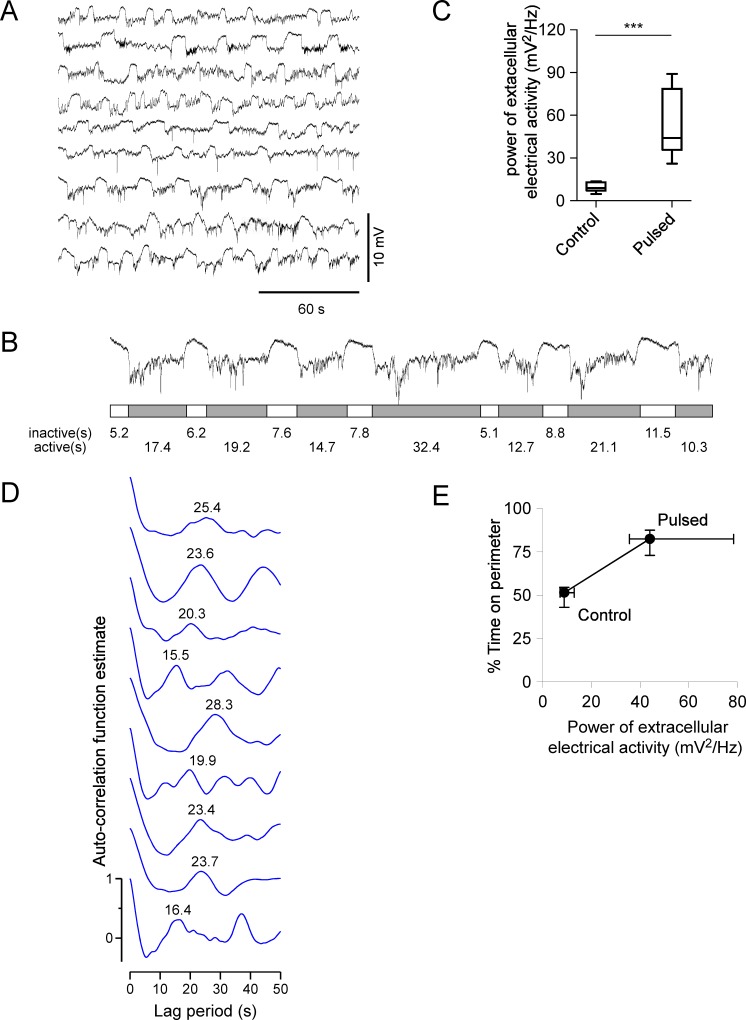
Pulsed light stimulation increased struggling activity of *w*^*1118*^ male flies. (A) Extracellular recordings from *w*^*1118*^ flies subjected to pulsed light stimulation for entire life cycle and 4 additional days since emergence. (B) Demonstration of the time duration calculation of inactive and active phases of the pulsed *w*^*1118*^ flies. The time duration of inactive or active phases were calculated with Clampfit 10 software (Molecular Devices). To calculate the total inactive (active) time duration, the inactive (active) time periods are summed. (C) Plot of power (mV^2^/Hz) vs control (n = 8) and pulsed (n = 9) *w*^*1118*^ flies. Asterisks (* or ***) indicate P < 0.05 or P < 0.001 by Kruskal-Wallis test. (D) Auto-correlation analysis of periodicities in pulsed flies. Values are the periodicity estimates for most recordings. (E) Correlation analysis between % TOP and power of struggling activity in control (n = 8 for both % TOP and power analysis) and pulsed (n = 8 for % TOP and n = 9 for power analysis) *w*^*1118*^ flies. Black dots and error bars indicate median, 25% and 75% percentile of values.

To determine whether pulsed light stimulation affected the CNS of *w*^*1118*^ flies, we examined the effects on the electrical activity which was associated with centrally-generated motor pattern. After pulsed light stimulation, fluctuations with increased amplitude were observed consistently in the recordings (Fig 4A) compared with *w*^*1118*^ controls under regular light/dark illumination (see Fig 3B). In addition, the two-phase alternation was seen consistently from fly to fly (Fig 4B). For individual flies, each active (inactive) time periods were summed and the ratio of total active time to total inactive time was calculated. For pulse-illuminated *w*^*1118*^ flies, the median of the ratio is 3.34 (IQR 2.93–5.16), which is higher than the ratio of CS flies (median 0.76, IQR 0.34–0.95), indicating that the duration of the relatively inactive phase in pulsed flies was shorter than that of active phase.

The broad spectral analysis indicates 0.01–0.1 Hz activities are the major components of our recordings. Therefore, the summation of the power spectrum in 0.01–0.1 Hz was calculated, and analyzed between flies. The power of the extracellular electrical activity increased in *w*^*1118*^ flies with pulsed light stimulation relative to the controls (*P* <0.001, Kruskal-Wallis test with Dunn’s multiple comparison) (Fig 4C). The auto-correlation analysis revealed a periodicity of the struggling activity in the range of 15.5–25.4 s which was consistently present in flies (Fig 4D). Therefore, pulsed light stimulation improved the struggling motor pattern in *w*^*1118*^ flies, measured as an increase in the power of extracellular electrical activity, which also demonstrated two-phase (inactive/active) alternation of activities.

### Increased power of extracellular electrical activity correlated with increased % TOP in *w*^*1118*^ flies with pulsed light stimulation

*w*^*1118*^ control flies displayed low power of extracellular electrical activity and low % TOP, whereas *w*^*1118*^ flies with pulsed light stimulation showed increased power and increased % TOP (Fig 4E). Thus, the enhancement of the electrical activity correlated with the increase of % TOP in *w*^*1118*^ flies.

### Normal periodic electrical activity was associated with genetic background

Without pulsed light treatment, CS flies showed periodic electrical activity while no similar fluctuations could be observed in *w*^*1118*^ flies (Fig 3). Autocorrelation function estimates were made to examine the periodicity of the recorded activity without pulsed light treatment. The analysis was conducted with a maximal lag period of 40 s, which covered the ~20 s periodicity of preliminary data in wild-type CS flies. Typically, with a lag period ranging from 0 to 40 s, autocorrelation function estimates drop from 1 (zero lag) to negative values, then gradually increase to the first peak, which indicates the periodicity of the episodic activity. Preliminary observations showed that almost all the recordings of CS flies without pulsed light treatment had the first peak autocorrelation estimates between 0.3 and 0.5, whereas no recording of *w*^*1118*^ flies without pulsed light treatment had an estimate greater than 0.3. Therefore, an estimate value of 0.3 was applied to classify the autocorrelation outcomes between CS and *w*^*1118*^, and all subsequent analyses of progeny flies including F1 and F10 without pulsed light treatment. Recording with autocorrelation estimate greater than 0.3 was considered as indicating the presence of rhythmic electrical activity associated with centrally-generated motor pattern, and lower than 0.3 as indicating the absence/reduction of rhythmic electrical/motor activity. Periodicity was measured from the lag period corresponding to the peak estimate.

**Fig 5:**
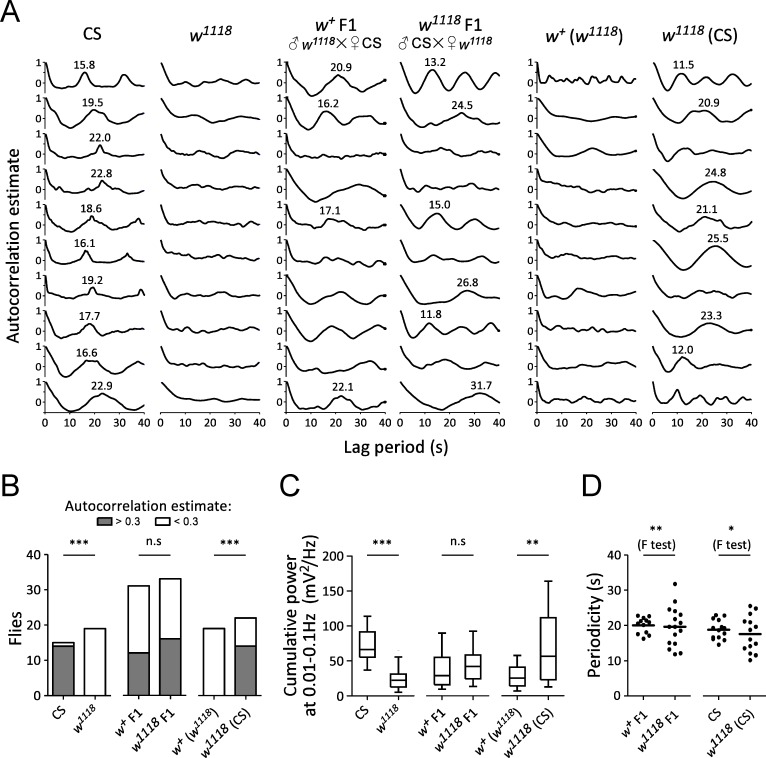
Contributions of the *w* gene and genetic background to rhythmic electrical activity without pulsed light stimulation. (**A**) Autocorrelation analysis of rhythmic electrical/struggling activity in CS, *w*^*1118*^ and their progeny without pulsed light treatment. Values are the peaks of lag period with estimate > 0.3, which indicates strong periodicity. (**B**) Proportional analysis of rhythmic electrical/motor activities with autocorrelation estimate > 0.3 (grey box) and < 0.3 (open box) between CS (n = 15) and *w*^*1118*^ (n = 19), and between *w*^*+*^ and *w*^*1118*^-carrying progeny (*w*^*+*^ F1: n = 31; *w*^*1118*^ F1: n = 33; *w*^*+*^ (*w*^*1118*^): n = 19; *w*^*1118*^ (CS): n = 22) without pulsed light treatment. *P* values are from Fisher’s exact tests. (**C**) Comparison of cumulative power amplitude at 0.01–0.1 Hz (mV^2^/Hz) between strains (CS: n = 15; *w*^*1118*^: n = 35; *w*^*+*^ F1: n = 17; *w*^*1118*^ F1: n = 19; *w*^*+*^ (*w*^*1118*^): n = 19; *w*^*1118*^ (CS): n = 19) without pulsed light treatment. *P* values are from Mann-Whitney tests. (**D**) Variance of periodicities between *w*^*+*^ and *w*^*1118*^-F1 (n = 12 and 16, respectively), and between CS (n = 14) and *w*^*1118*^ (CS) (n = 14) without pulsed light treatment. Periodicities in recordings with autocorrelation estimate > 0.3 were used for analysis. *P* values are from F tests.

Without pulsed light treatment, periodic electrical activity associated with episodic motor activity in CS flies displayed peak estimates greater than 0.3 in 93% (14/15) of the preparations, whereas *w*^*1118*^ showed peak estimate > 0.3 in 0% (0/19) of the preparations (Fig 5A). The rhythmic electrical/motor activity occurred more frequently in CS flies than *w*^*1118*^ (*P* < 0.0001, Fisher’s exact test) (Fig 5B).

To examine whether the *w* gene contributes to the occurrence of this periodic electrical/ motor activity without pulsed light treatment, male progeny (F1) containing *w*^*+*^ or *w*^*1118*^ were tested. Recordings of *w*^*+*^ F1 flies showed peak estimate > 0.3 in 39% (12/31) preparations, while recordings of *w*^*1118*^ F1 flies displayed peak estimate > 0.3 in 48% (16/33) preparations. The percentages of flies showing episodic electrical activity which was associated with motor activity were statistically the same between *w*^*+*^ F1 and *w*^*1118*^ F1 flies (*P* = 0.4607, Fisher’s exact test) (Fig 5B), indicating the dissociation between *w*^*+*^ and the occurrence of rhythmic motor activities.

To confirm the relation between *w*^*+*^ and the occurrence of the rhythmic electrical activity which was associated with a centrally-generated motor pattern, *w*^*+*^ (*w*^*1118*^) and *w*^*1118*^ (CS) flies generated by serial backcrossing between CS and *w*^*1118*^ without pulsed light treatment were tested. *w*^*+*^ (*w*^*1118*^) displayed episodic electrical/motor activity with peak estimate > 0.3 in 0% (0/19) preparations, whereas *w*^*1118*^ (CS) showed peak estimate > 0.3 in 64% (14/22) flies. Thus rhythmic electrical/motor activity was observed with a higher percentage in *w*^*1118*^ (CS) than that in *w*^*+*^ (*w*^*1118*^) flies (*P* < 0.0001, Fisher’s exact test) (Fig 5B). These results indicate that genetic background but not *w*^*+*^ was strongly associated with the occurrence of rhythmic electrical/motor activity in wild-type flies.

The statistical difference of cumulative power amplitude at 0.01–0.1 Hz between fly strains without pulsed light treatment was examined as well (Fig 5C). The cumulative power amplitude in CS flies (median 66.3 mV^2^/Hz, interquartile range (IQR) 58.4–94.0 mV^2^/Hz, n = 15) was higher than that in *w*^*1118*^ (median 22.4 mV^2^/Hz, IQR 12.8–31.2 mV^2^/Hz, n = 35) (*P* < 0.0001, Mann-Whitney test) (Fig 5C). The cumulative power in *w*^*+*^ F1 (median 29.0 mV^2^/Hz, IQR 16.0–55.3 mV^2^/Hz, n = 17) was statistically the same as that in *w*^*1118*^ F1 (median 42.1 mV^2^/Hz, IQR 24.3–58.5 mV^2^/Hz, n = 19) (*P* = 0.3749, Mann-Whitney test) (Fig 5C). However, the cumulative power in *w*^*+*^ (*w*^*1118*^) (median 25.4 mV^2^/Hz, IQR 14.1–41.1 mV^2^/Hz, n = 19) was lower than that in *w*^*1118*^ (CS) (median 56.8 mV^2^/Hz, IQR 22.9–112.0 mV^2^/Hz, n = 19) (*P* = 0.0051, Mann-Whitney test) (Fig 5C). Therefore, without pulsed light treatment, the wild-type genetic background contributed to a high cumulative power amplitude. These data support the association between wild-type genetic background and the occurrence of periodic electrical activity in wild-type flies. However, similar to boundary preference reported before [32], *w*^*+*^ was minimally involved in this episodic electrical/motor activity.

### *w*^*+*^ modulated the consistency of periodicity

Although the occurrence of periodic electrical activity and centrally-generated motor pattern was strongly associated with the wild-type genetic background, there is a possibility that the consistency of the activity across individual flies is modulated by *w*^*+*^. The periodicities of activity observed in *w*^*+*^ F1 and *w*^*1118*^ F1 flies without pulsed light treatment were examined. Both flies carried the same genetic background on second and third chromosomes but different X chromosome and *w* alleles. Periodicities in *w*^*+*^ F1 (mean ± SD: 20.0 ± 2.2 s, n = 12) showed smaller variance than those in *w*^*1118*^ F1 (19.7 ± 5.6 s, n = 16) (*P* = 0.0044, F test), although there was no statistical difference of average periodicities between *w*^*+*^ F1 and *w*^*1118*^ F1 flies (Fig 4D). Also, periodicities of activity observed in CS and *w*^*1118*^ (CS) flies, which carried nearly identical genetic background and different *w* alleles, were examined. Without pulsed light stimulation, periodicities in CS (18.8 ± 2.7 s, n = 14) displayed smaller variance than those in *w*^*1118*^ (CS) flies (17.6 ± 5.1 s, n = 14) (*P* = 0.0315, F test) with no difference of averages between flies (Fig 4D). Therefore, a higher consistency of periodicity was observed in *w*^*+*^-carrying flies.

## Discussion

We report here that *w*^*1118*^ flies generated motor activity in response to the chronic pulsed light stimulation, not only at the level of behavior with improved boundary preference and increased travel distance in a locomotor assay, but also at the level of electrical activity which was associated with a centrally-generated motor pattern showing enhanced periodic electrical/struggling activity. Additionally, the enhancement of the struggling motor pattern correlated with the increase of % TOP during locomotion. Genetic analysis indicates that the wild-type genetic background contributes largely to the generation of periodic motor activity, and that *w* modulates the consistency of periodicities between individuals.

In the current study, one of the most prominent observations is the occurrence of a two-phase alternation of extracellular electrical activity induced by pulsed light stimulation in the brain of *Drosophila* mutant *w*^*1118*^. The induced two-phase alternation displays a periodicity around 20 s with the duration of inactive phase shorter than active phase instead of longer inactive phase accompanied with shorter active phase directly from the pulsed light stimulation protocol, suggesting that these flies have integrated the pulsed light stimulation and generated a stabilized and consistent struggling pattern rather than simply generating responses reflecting the cycle of pulsed light stimulation (5 s ON, 15 s OFF). The critical change in the pulsed *w*^*1118*^ flies is the organized activity with the enhancement of inactive/active phase alternation, which is largely missing in *w*^*1118*^ flies with random activity under regular illumination. Most biological rhythms, although affected by external stimulation, are generated endogenously [41]. It is likely that the sensory stimulation by light enhances the synchronous neural activity, which is likely related to neuroplasticity. The periodic inactivity in the centrally-generated motor pattern indicates that flies are able to rhythmically reduce the activity from continuous struggling activity by presenting an inactive/active alternation. Such a reduction on activity would suppress or remove the sporadic and spontaneous activity, and reinforce the rhythmicity. Conceivably, periodic transition between relatively inactive and active phases would increase the efficiency in many biological processes such as efficient energy consumption.

The behavioral consequences induced by pulsed light stimulation in the locomotor assay indicated that the increased locomotor activity on the perimeter and increased travel distance require a high degree of locomotion coordination. Defects in locomotion coordination resulted in reduced speed and path length in chordotonal organ mutants (cho), called *atonal (ato)*, in *Drosophila* larva [42, 43]. In mammals, severe motor coordination deficit reduced edge preference in Tenascin-R-deficient mice [44]. With pulsed light treatment, *w*^*1118*^ flies display improved locomotion coordination and are likely highly concentrated on the exploratory task by spending more time on the perimeter and traveling longer distance [45], which are the typical locomotor characteristics in wild-type flies [32].

Even though it has been shown that sensory stimulation can enhance motor performance, the relationship between brain plasticity and the improvement of motor performance is still unclear. It has been suggested that stimulation leads to structural and functional remodeling in brain [46–48] and the reorganization of neural connections could be activated by multiple plasticity mechanisms such as the expression or release of activity-dependent neurotrophins [49–51], which might promote enhanced coordination and other circuit functions.

In recent years, repetitive stimulation has attracted attention for stroke therapy and repetitive stimulations with electrical pulses have been shown to improve sensorimotor tasks in human adults [7, 8, 52]. Similar to repetitive electrical stimulation with electrical pulses, pulsed light treatment in our study has a major advantage that the treatment is applied passively and no active cooperation or even attention is required when the unattended stimulation takes place [7].

In summary, this study shows that adult *w*^*1118*^ flies could generate a motor activity response to chronic sensory stimulation, both at the level of behavior (e.g. boundary preference and travel distance in a locomotor assay) and at the level of electrical activity which was associated with a centrally-generated motor pattern (struggling activity). The mechanisms of brain plasticity responsible for this response are still unknown, however, we have shown that they have a genetic basis and thus could be addressed by the sophisticated techniques afforded by a *Drosophila* model.

## Supporting Information

S1 Video Relation between rhythmic struggling activities and extracellular electrical activity in wild-type CS fly. (MP4)
